# Supplementary material for: Polar and phase domain walls with conducting interfacial states in a Weyl semimetal MoTe2
Source: Nat Commun. 2019 Sep 16;10:4211. doi: 10.1038/s41467-019-11949-5 (PMC6746811; doi:10.1038/s41467-019-11949-5)
Supplement: Supplementary file 1 — Supplementary Information [file 41467_2019_11949_MOESM1_ESM.pdf]

Supplementary Information

Polar and phase domain walls with conducting interfacial states in a Weyl semimetal $MoTe_2$

Huang *et al.*

Supplementary Note 1 The origin of the uncompensated dipole in T_d-$MoTe_2$

In this section, we explain the polar distortion in semimetallic $MoTe_2$ from the crystallographic point of view. The geometric structure of centrosymmetric 1T'-I $MoTe_2$ with + + interlayer pattern is illustrated in Supplementary Figure 1a. Each Mo ion (blue spheres) sits in a distorted Te octahedron. Because of the Mo-Mo metallic bonding, Mo ions shift off the center of the distorted Te octahedra. Green arrows indicate the $\pm z$ displacement of Mo ions away from the average z positions of the neighboring Te ions. Since the top and bottom Te layers (yellow and red spheres) are confined by space inversion symmetry (the inversion center located at the Mo-Mo bond marked by x), the $\pm c$ dipole moment is cancelled out. The intrinsic difference between 1T' and T_d arises from the interlayer gliding of the structure. As long as the interlayer gliding is arranged in either + - or - + pattern, it will break the inversion symmetry while any additional vertical displacement is not required as shown in Supplementary Figure 1b. The non-centrosymmetric structure is also clear when tracking the Te-Te interlayer bonding outlined by orange and purple dashed lines (Supplementary Figure 1b). The absence of any inversion center induced by the interlayer gliding correlated with the fact that $\pm c$ directions are geometrically non-equivalent. It does not necessarily accompany the existence of a net dipole moment, in the sense that assuming each P or M layer is as rigid and symmetric as that of 1T' (Supplementary Figure 1a inset). Thus, any interlayer gliding is not sufficient to explain the polar origin in the T_d phase.

The inset of Supplementary Figure 1b demonstrates that the polar distortion is, in fact, caused by the local asymmetric bonding environment between the top Te (green and dark blue spheres) and bottom Te (yellow and red spheres) ions around the Mo ions. Because of the broken inversion symmetry, now the top and bottom Te are symmetry independent as indicated by different colors. Consequently, it gives additional vertical degree of freedom of those Te sites and leads to a net dipole moment along the c axis. The estimation of the net dipole moment based on the T_d structure [ref. 34 in the main text] is 3.6×10^{11} e^{-} cm^{-2} (= 0.058 μC cm^{-2}). Note that a recent report on monolayer $MoTe_2$ with the d1T trimerized structure can achieve 0.68 μC cm^{-2} from the DFT calculations [ref. 38 in the main text]. As discussed above, the asymmetric Te bonding environment of the T_d phase is the reason that moves the average negative center away from the Mo-Mo center even though the magnitude is almost negligible compared with traditional ferroelectric perovskite (three orders smaller than $BaTiO_3$). Despite the fact that

$MoTe_2$ is semimetal, the charge distribution is expected to be highly anisotropic in the van der Waals layered structure. Therefore, the dipole-dipole interaction in the polarization direction may not be screened as a similar mechanism proposed in ferroelectric metal $LiOsO_3$ [ref. 23 in the main text]. Finally, we note that two distinct surfaces of $MoTe_2$ at low temperatures have been reported in literatures [refs. 29-30 in the main text], and they correspond to the T_d↑ and T_d↓ in this work.

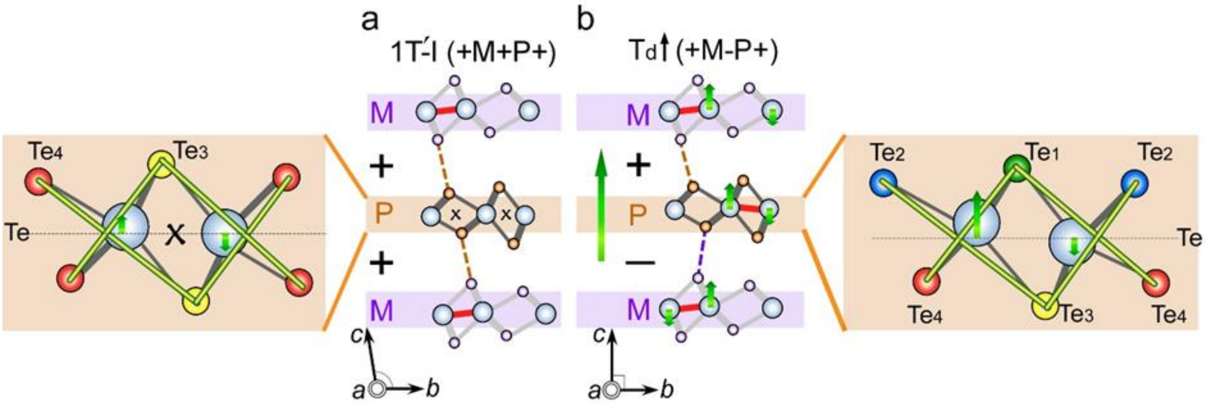

Supplementary Figure 1. Schematics view of the macroscopic polarization in the T_d phase. **a**, 1T'-I and **b**, T_d↑ phase of $MoTe_2$. Mo, blue; Te of P-layer, orange; Te of M-layer, purple. The insets show the bonding environments of the P layers. A net out-of-plane dipole moment can be induced in the T_d phase as a result of the slightly different Te environments on the top and bottom layers. Horizontal dotted lines shown in the insets indicate the average *z* positions of Te octahedra. Green arrows correspond to the displacements of Mo ions along the *c* axis. The symbol *x* marks the inversion center.

Supplementary Note 2: The persistence of phase coexistence in the presence of mechanical constraints

As discussed in Figure 1 in the main text, the *b*-*c* angle in the monoclinic 1T' phase is associated with a mechanical glide of the layers. The macroscopic shear deformation during the phase transition involves the accumulated glide of the top layer relative to the bottom one by an amount which is proportional to the twin thickness along *c* axis. In the case of freely suspended or uncapped samples, the shear deformation can occur with no further energy cost, so that the entire sample can complete the structural transition into a single phase either of 1T' or T_d, depending on temperature. However, in a constrained situation such as a capped specimen, mechanical constraints will apply restoring forces that tend to resist the glide of the topmost layer from its initial position. For example, in this work, our cross-section TEM specimens were made by clamping two silicon slabs and one $MoTe_2$ thin plate together with sides facing each other. Experimentally, we always observe asymmetric 1T'-I and 1T'-II twin fractions (Figure 2b and Figure 2e in the main text) at room temperature, thus the 1T' to T_d phase transition entails a shear deformation proportional to the thickness difference between the 1T'-I and 1T'-II domains. The resulting behavior depends on the strength of the elastic restoring forces. If these are strong enough that the net glide is almost forbidden, some fraction of 1T' phase should always remain even below T_c as seen in this work. For instance, if the twin domain fractions are x and $1-x$ for the initial 1T'-I and 1T'-II phases respectively, with $x < ½$, then a fraction of $2x$ can transform to T_d while leaving a fraction $1-2x$ of 1T'-II remaining. Therefore, the enhanced phase coexistence observed in the capped samples can be understood as an effect of mechanical constraints that tend to prevent the layer glide. In other words, we freeze in the coexistence of 1T' and T_d phases in a quasi-periodic superlattice-like arrangement in the case of the capped specimen geometry at 80 K.

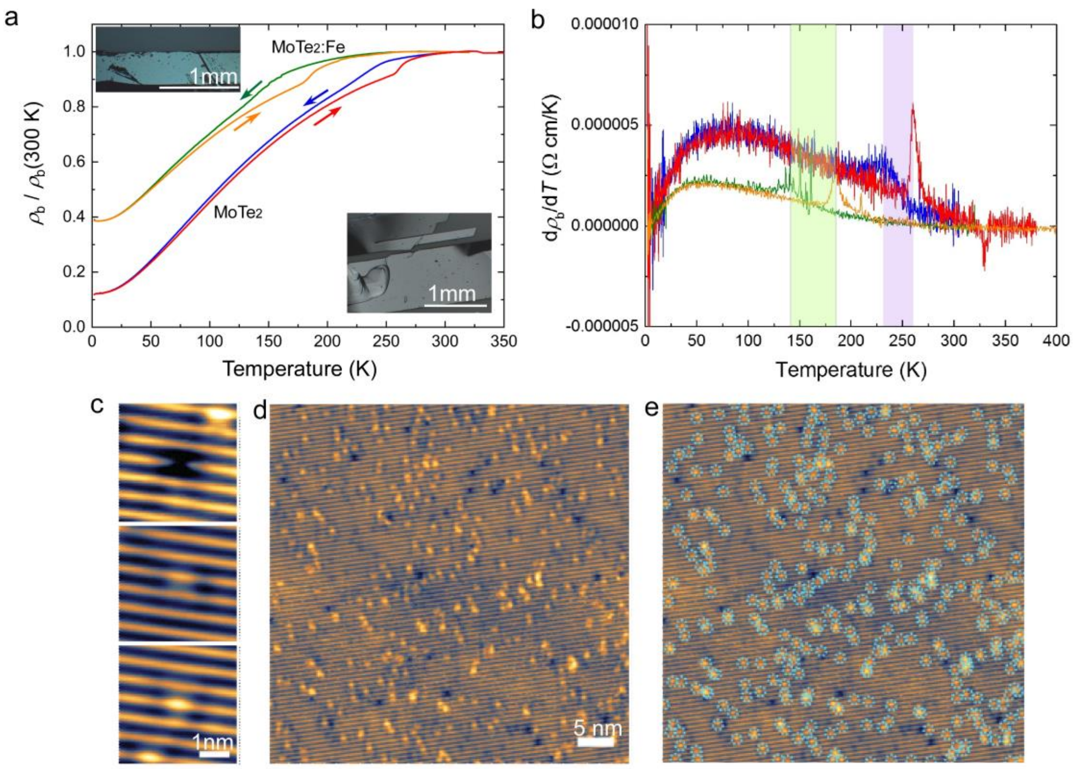

Supplementary Figure 2. Chemically tunable WSM transition. a, Temperature dependence of the *ab*-plane electrical resistivity of 1T'-$MoTe_2$ and 1T'-$MoTe_2$:Fe single crystals at ambient pressure. Measurements were carried out with the electric current fixed along the *b* crystallographic axis. Insets show photographic images of crystals. Scale bar, 1 mm. The 1T' to T_d polar transition can be identified from the sudden decrease of resistance during cooling (blue and green curves) and the abrupt increase of resistance during warming (red and orange curves). Both resistivity curves show metallic behavior with a thermal hysteresis setting in below room T. **b**, Temperature derivative of resistivity, dρ(T)/dT. Anomalous hysteresis loops are observed at 260-230 K in 1T'-$MoTe_2$ and 180-135 K in 1T'-$MoTe_2$:Fe single crystals. The phase transition can be tuned to lower temperature with Fe doping. **c,** Topographies of three typical defects found in 1T'-$MoTe_2$:Fe samples with scan parameters as -0.3V, 100 pA: top, a depression in the middle of a zigzag; middle, a protrusion connecting two zigzags; bottom, a protrusion in the middle of a zigzag. The first defect with the depression is identified as a Te vacancy while the bottom two defects with protrusion features are considered as a single Fe impurity as marked in (e). Scale bar: 1 nm. **d,e,** Estimation of Fe concentration is performed on a STM topography of 50 nm lateral size shown in (**d**) with scan parameters as -0.3V, 200 pA. The resulting estimation of the real composition of Fe impurities is ~1.06% (359 Fe atoms in a 50×50 nm^2 square area).

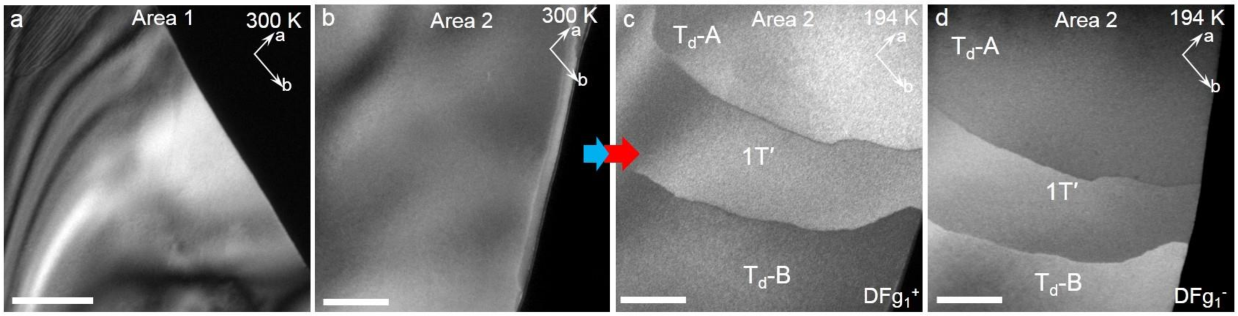

Supplementary Figure 3. In-plane view of phase coexistence during a warming cycle. a, DF-image showing no domain at room temperature in the nearby area of Figure 4a in the main text. Scale bar: 500 nm. **b**, DF-image showing no domain at room temperature in another area. During cooling down to 80 K and the following warming up process, the coexistence of 1T', T_d↑ and T_d↓ domains was found at 194 K. **b,c,** DF-image selecting $g_1^+ = (1, 2, \bar{1})$ spot in (**b**) and $g_1^- = (\bar{1}, \bar{2},1)$ spot in (**c**), showing the appearance of 1T' domain in the T_d matrix during the warming process. The reversed contrasts of T_d↑ and T_d↓ are associated with a space-inversion breaking in the T_d phase while the contrast of the central 1T' domain remains. Cycling the same TEM specimen through T_c leads to a completely different polar domain patterns, indicating that the domain formation is not simply due to pinning by disorder such as chemical defects or dislocations. We note that 1T' shows up at 194 K which is lower than the data from the T_c obtained in the transport measurement (shown in Supplementary Figure 2a) with a large coexistence temperature window of more than 65 K. On the other hand, a rather sharp transition has also been observed in a relatively thin specimen as shown in Supplementary Figure 4. Scale bar, 500 nm.

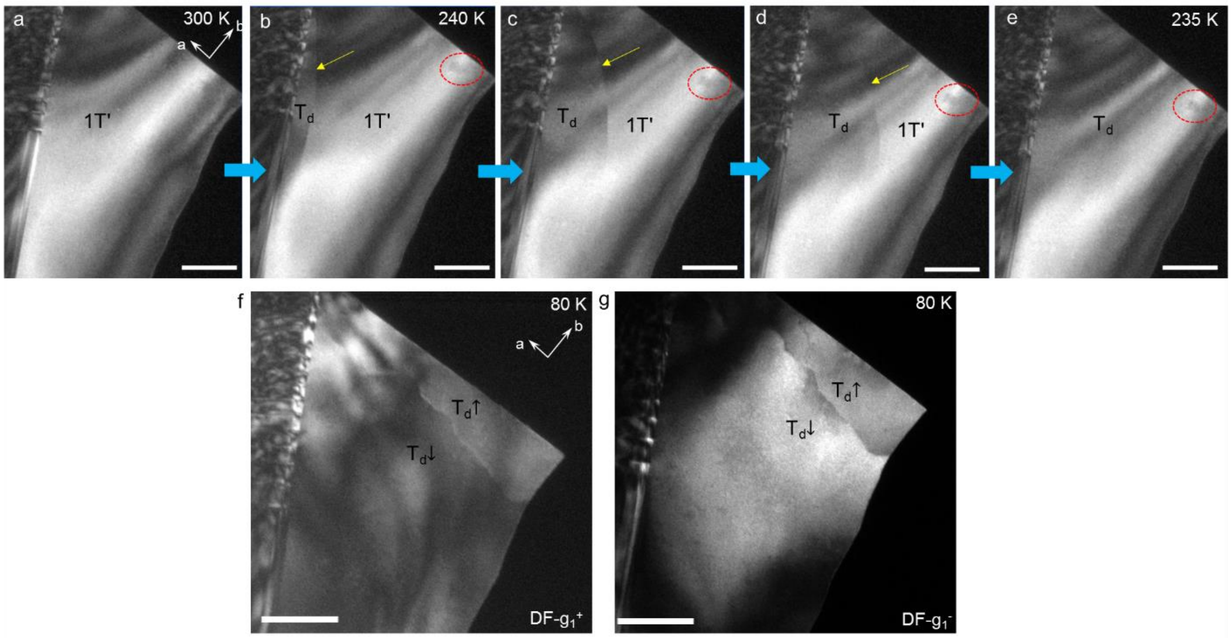

Supplementary Figure 4 In-plane view of single 1T' to single T_d domain evolution. a-e, Sequential DF-images of in-situ cooling in moderately thin specimen (thickness < 30 nm), showing a rather sharp transition at ~260 K with a small temperature hysteresis (~5 K). It is consistent with the anisotropic phase evolution with a preferred layer-by-layer shearing path as discussed in the main text. A new T_d domain nucleates and grows from the left to the right, leading to a bright to dark change in the domain contrast. The yellow arrow indicates the phase domain walls between the 1T' and T_d phases. In term of the electron beam effect, if we start with 1T' phase, a beam-induced damaging instead of phase switching occurred as indicated by red circles. No phase transition is observed at room temperature. **f,g**, DF-images of beam induced T_d domains after a beam exposure at the specimen edge. A new T_d domain appears at the edge. Both T_d domains show reversed contrasts when selecting g_1^+ spot (**f**) and g_1^- spot (**g**) for imaging at 80 K. We found that the e^- beam induced domains only occur in the T_d phase and not in the 1T' phase. The nucleated polar DWs tend to be parallel to the *a* axis. Note that the assignments of T_d↑ and T_d↓ is only for simplicity and needs further confirmation by measurements such as atomic-resolved STEM imaging. Scale bar, 500 nm.

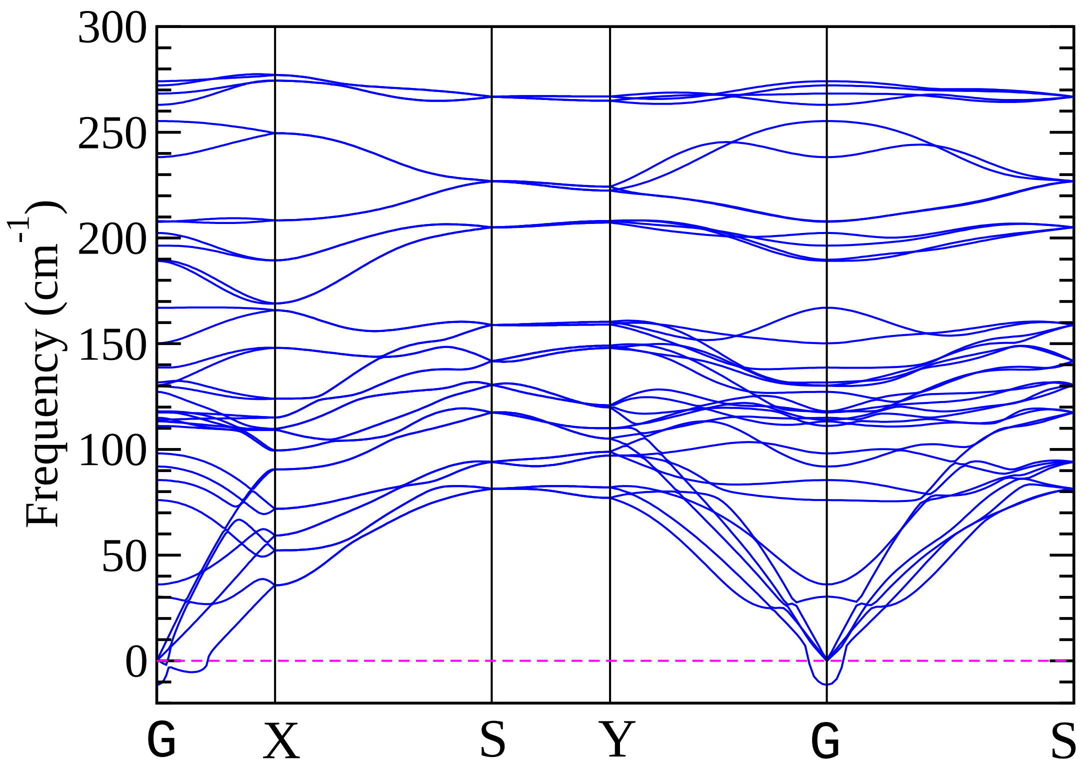

Supplementary Figure 5 Phonon frequencies of the T_0 phase calculated along the high-symmetry directions of momentum space. Two instabilities can be observed in the phonon band structure: (1) An unstable polar phonon mode present at the Γ point causing interlayer displacement of alternating $MoTe_2$ layers, and (2) an unstable acoustic mode along the Γ-X direction indicating the elastic instability present in the T_0 phase.

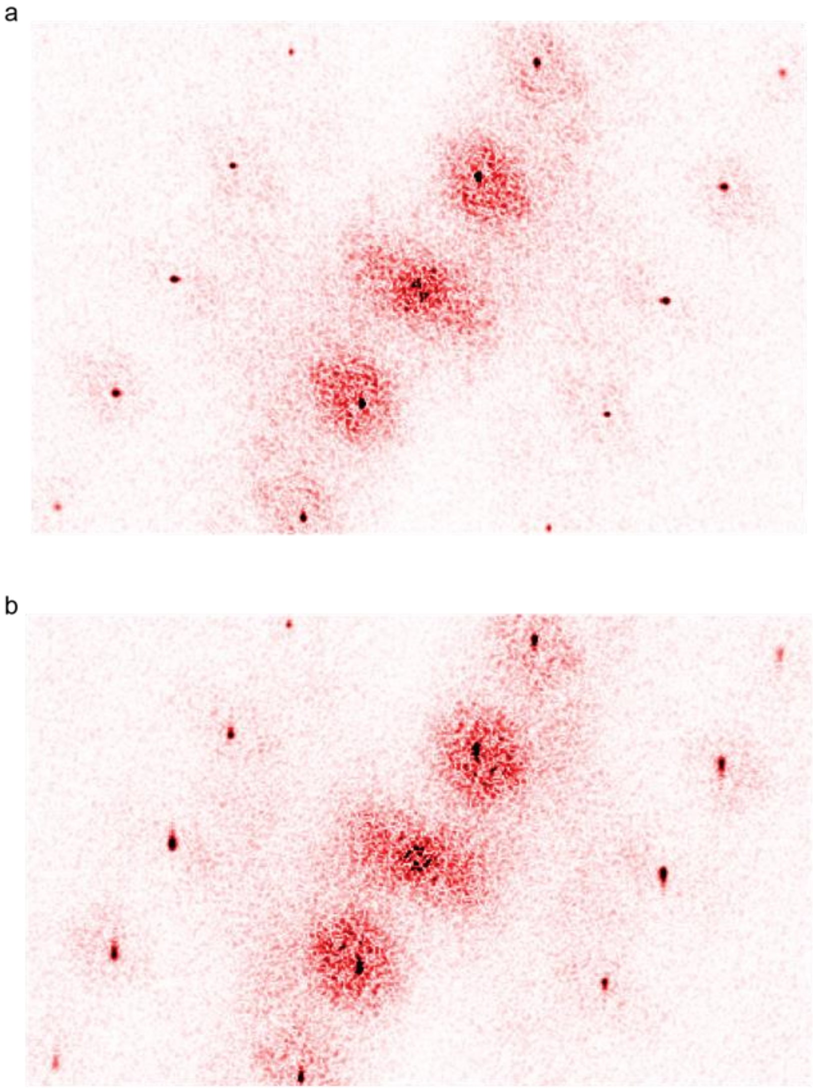

Supplementary Figure 6 Quasiparticle interference patterns at 77 K. We have tried to get a meaningful difference of quasiparticle interference pattern from multiple domains, however, all measurements showed similar results within our system's resolution or at the measurement temperature. **a,b,** Two representative patterns obtained from two nearby domains. There are clear 2×1 superlattice peaks appearing as a rectangular pattern of black dots. Although long-range featured near the center point differ in detail from different domains, we attribute the change to the effect of different atomic defects such as Fe dopants. The observed pattern near the center is more diffuse than the reported patterns of $MoTe_2$ [ref. 29 in the main text], which supports the effect of dopants in our measurement.

Supplementary Table 1: DFT+U optimized crystal parameters.

$MoTe_2$	Space group	Lattice parameters (Å)	Cell angles (degrees)
T_d↑/T_d↓	31	a = 6.329, b = 3.450, c = 13.485	α = 90°, β = 90°, γ = 90°
1T'-I	11	a = 6.329, b = 3.450, c = 13.485	α = 90°, β = 86.4, γ = 90°
1T'-II	11	a = 6.329, b = 3.450, c = 13.485	α = 90°, β = 93.6, γ = 90°
T_0	62	a = 6.329, b = 3.450, c = 13.485	α = 90°, β = 90°, γ = 90°

Direct atomic coordinates:

T_0 phase

	x	*y*	*z*
Mo	0.11349	0.25000	0.00690
Mo	0.25103	0.75000	0.50696
Mo	0.75096	0.75000	0.99309
Mo	0.61343	0.25000	0.49305
Te	0.50373	0.25000	0.10509
Te	0.00793	0.75000	0.15199
Te	0.50704	0.75000	0.34798
Te	0.00286	0.25000	0.39494
Te	0.36073	0.75000	0.89490
Te	0.85653	0.25000	0.84799
Te	0.35740	0.25000	0.65202
Te	0.86158	0.75000	0.60507

T_d↑(T_d↓) phase

	x	*y*	*z*
Mo	0.14865 (0.07433)	0.25000 (0.25000)	0.00709 (0.00641)
Mo	0.21580 (0.29012)	0.75000 (0.75000)	0.50714 (0.50645)
Mo	0.79010 (0.71579)	0.75000 (0.75000)	0.99355 (0.99286)
Mo	0.57435 (0.64866)	0.25000 (0.25000)	0.49360 (0.49291)
Te	0.54017 (0.46562)	0.25000 (0.25000)	0.10525 (0.10506)
Te	0.04564 (0.97440)	0.75000 (0.75000)	0.15258 (0.15174)
Te	0.47442 (0.54566)	0.75000 (0.75000)	0.34826 (0.34742)
Te	0.96563 (0.04018)	0.25000 (0.25000)	0.39494 (0.39475)
Te	0.39882 (0.32427)	0.75000 (0.75000)	0.89490 (0.89471)
Te	0.89001 (0.81881)	0.25000 (0.25000)	0.84821 (0.84737)
Te	0.31883 (0.39003)	0.25000 (0.25000)	0.65263 (0.65179)
Te	0.82429 (0.89884)	0.75000 (0.75000)	0.60529 (0.60510)

1T'-I (1T'-II) phase

	x	*y*	*z*
Mo	0.11221 (0.11066)	0.25000 (0.25000)	0.00726 (0.00631)
Mo	0.25428 (0.25180)	0.75000 (0.75000)	0.50628 (0.50723)
Mo	0.75177 (0.75429)	0.75000 (0.75000)	0.99276 (0.99370)
Mo	0.61063 (0.61215)	0.25000 (0.25000)	0.49370 (0.49276)
Te	0.51773 (0.48836)	0.25000 (0.25000)	0.10500 (0.10532)
Te	0.02787 (-0.00714)	0.75000 (0.75000)	0.15274 (0.15235)
Te	0.49236 (0.52739)	0.75000 (0.75000)	0.34765 (0.34728)
Te	-0.01207 (0.01729)	0.25000 (0.25000)	0.39464 (0.39502)
Te	0.34624 (0.37660)	0.75000 (0.75000)	0.89500 (0.89468)
Te	0.83615 (0.87208)	0.25000 (0.25000)	0.84728 (0.84767)
Te	0.37254 (0.33659)	0.25000 (0.25000)	0.65233 (0.65271)
Te	0.87700 (0.84664)	0.75000 (0.75000)	0.60535 (0.60498)
