# Supplementary material for: Polar and phase domain walls with conducting interfacial states in a Weyl semimetal MoTe2
Source: Nat Commun. 2019 Sep 16;10:4211. doi: 10.1038/s41467-019-11949-5 (PMC6746811; doi:10.1038/s41467-019-11949-5)
Supplement: Supplementary file 3 — Description of Additional Supplementary Files [file 41467_2019_11949_MOESM3_ESM.pdf]

Description of Additional Supplementary Files

Supplementary Movie 1: In-situ movie of the polar DWs motion in $MoTe_2$ at 80 K right after a focused electron beam of TEM, which corresponds to Fig. 5c in the main text.

Supplementary Movie 2: In-situ movie of the reversible switching process of polar DW motion after defocusing electron beam.
